# Surgery versus radiosurgery for vestibular schwannoma: Shared decision making in a multidisciplinary clinic

**DOI:** 10.1093/noajnl/vdad089

**Published:** 2023-07-19

**Authors:** Francesca Colombo, Helen Maye, Scott Rutherford, Andrew King, Charlotte Hammerbeck-Ward, Gillian A Whitfield, Catherine McBain, Rovel Colaco, Helen Entwistle, Andrea Wadeson, Simon Lloyd, Simon Freeman, Omar N Pathmanaban

**Affiliations:** Department of Neurosurgery, Manchester Centre for Clinical Neurosciences, Salford Royal NHS Foundation Trust, Manchester, UK; Department of Neurosurgery, Manchester Centre for Clinical Neurosciences, Salford Royal NHS Foundation Trust, Manchester, UK; Department of Neurosurgery, Manchester Centre for Clinical Neurosciences, Salford Royal NHS Foundation Trust, Manchester, UK; Department of Neurosurgery, Manchester Centre for Clinical Neurosciences, Salford Royal NHS Foundation Trust, Manchester, UK; Department of Neurosurgery, Manchester Centre for Clinical Neurosciences, Salford Royal NHS Foundation Trust, Manchester, UK; Department of Neuro-Oncology, The Christie Hospital NHS Foundation Trust, Manchester, UK; Department of Neuro-Oncology, The Christie Hospital NHS Foundation Trust, Manchester, UK; Department of Neuro-Oncology, The Christie Hospital NHS Foundation Trust, Manchester, UK; Department of Neurosurgery, Manchester Centre for Clinical Neurosciences, Salford Royal NHS Foundation Trust, Manchester, UK; Department of Neurosurgery, Manchester Centre for Clinical Neurosciences, Salford Royal NHS Foundation Trust, Manchester, UK; Department of Neurosurgery, Manchester Centre for Clinical Neurosciences, Salford Royal NHS Foundation Trust, Manchester, UK; Department of Neurosurgery, Manchester Centre for Clinical Neurosciences, Salford Royal NHS Foundation Trust, Manchester, UK; Geoffrey Jefferson Brain Research Centre, Manchester Centre for Clinical Neurosciences, Division of Neuroscience, School of Biological Sciences, Faculty of Biology, Medicine, and Health, University of Manchester and Manchester Academic Health Sciences Centre, Manchester, UK

**Keywords:** patient counseling, skull base, shared decision making (SDM), vestibular schwannoma (VS)

## Abstract

**Background:**

Our neurosurgical unit adopted a model of shared decision-making (SDM) based on multidisciplinary clinics for vestibular schwannoma (VS). A unique feature of this clinic is the interdisciplinary counseling process with a surgeon presenting the option of surgery, an oncologist radiosurgery or radiotherapy, and a specialist nurse advocating for the patient.

**Methods:**

This is a retrospective cohort study. All new patients seen in the combined VS clinic and referred from the skull base multidisciplinary team (MDT) from beginning of June 2013 to end of January 2019 were included. Descriptive statistics and frequency analysis were carried out for the full cohort.

**Results:**

Three hundred and fifty-four patients presenting with new or previously untreated VS were included in the analysis. In our cohort, roughly one-third of patients fall into each of the treatment strategies with slightly smaller numbers of patients undergoing surgery than watch, wait and rescan (WWR) ,and SRS (26.6% vs. 32.8% and 37.9%, respectively).

**Conclusion:**

In our experience, the combined surgery/oncology/specialist nurse clinic streamlines the patient experience for those with a VS suitable for either microsurgical or SRS/radiotherapy treatment. Decision-making in this population of patients is complex and when presented with all treatment options patients do not necessarily choose the least invasive option as a treatment. The unique feature of our clinic is the multidisciplinary counseling process with a specialist nurse advocating and guiding the patient. Treatment options are likely to become more rather than less complex in future years making combined clinics more valuable than ever in the SDM process.

**Key Points**

1. The combined clinic offers a unique experience of shared decision-making for patients with vestibular schwannoma.
2. Treatment options are becoming more complex making combined clinics more valuable than ever.
3. Some patients with growing tumors elect to continue to observe.

**Importance of the Study**

The combined skull base and clinical oncology clinic for vestibular schwannoma features access to a skull base surgeon and neuro-oncologists in a single appointment along with skull base specialist nurses and audiology. A unique feature of this clinic is the interdisciplinary counseling process with a surgeon presenting the case for surgery, an oncologist for radiosurgery or radiotherapy, and a specialist nurse advocating for the patient. Patient decision-making—empowered by the relevant information and support—is unique to each person and their current life situation and is more complex than may have been appreciated. Some patients with growing tumors elect to continue watching and waiting and a sizable group continue to elect surgery rather than radiosurgery.

Shared decision-making (SDM) is a joint process in which healthcare professionals work together with patients to reach a decision about care.^1^ Treatment options for complex conditions continue to increase, and the SDM model helps patients, families, and healthcare professionals to weigh the risks and benefits of each option to choose the most appropriate treatment.^2^ In 2010, the Health Foundation in the UK commissioned the MAGIC (Making Good Decisions in Collaboration) program in order to integrate SDM into primary and secondary care using quality improvement methods.^3^ NICE guidelines about SDM have published in 2021.^1^ Our neurosurgical unit adopted a model of SDM based on multidisciplinary clinics for benign but complex low-volume pathology including vestibular schwannoma (VS).

VSs are benign tumors originating from the eighth cranial nerve.^4^ They constitute up to 8% of intracranial tumors,^5^ and have an incidence between 3 and 5 per 100 000 per year.^6^ Only about 30% of VS grow enough over time to require intervention.^7–11^ Up until 1992 in Manchester every patient referred with a VS underwent surgery.^12^ Over the last 3 decades treatment goals in the management of this benign tumor have changed with emphasis now placed more on facial nerve preservation and attempts at hearing preservation rather than on removal of the tumor at all costs. Over the same time frame, radiosurgery with the gamma knife or Linear accelerator-based platforms became an increasingly recognized treatment option which has now become part of the standard practice in skull base surgeons’ armamentarium. In those with sporadic VSs 3 major treatment pathways exist namely watch, wait and rescan (WWR), radiotherapy/radiosurgery, and microsurgery.^13–16^

The Manchester Center for Clinical Neurosciences covers a tertiary catchment population of > 3 million patients in greater manchester as well as complex national and international referrals for review by the skull base multidisciplinary team (MDT). This includes > 200 new VS patients per year. All new referrals to the skull base service are discussed in the skull base MDT meeting carried out by neurosurgery, otolaryngology (ENT), neuro-oncology (radiotherapy), and radiology. This was established in 2009. Patients with VS suitable for observation are directly seen by a skull base surgeon and advised to adopt WWR. Those that we would recommend treatment for, or those close to a risk boundary with further growth with an obvious preferred treatment modality due to tumor or patient-specific factors are referred to the specific clinic, whilst patients amenable to more than one treatment option are referred to the combined VS clinic. The combined skull base and clinical oncology clinic for VS was set up in 2013 featuring access to neurosurgery, ENT, and neuro-oncologists in a single appointment along with skull base specialist nurses (SN) and audiology. A unique feature of this clinic is the interdisciplinary counseling process with a surgeon presenting the case for surgery, an oncologist for radiosurgery or radiotherapy, and a specialist nurse advocating for the patient and helping them to ask relevant questions and gain the relevant information to come to a shared decision with the team. Prior to this information leaflets have been provided to the patient. Although some surgeons are dual-trained in SRS and surgery, all SRS treatments in Manchester are currently delivered by radiation oncologists.

The aims of this study are to define the characteristics of the patient group referred to the combined clinic by the Manchester skull base MDT, to determine the treatment decisions reached by patients following the combined clinic consultation and to describe an interdisciplinary multidisciplinary clinic approach for SDM in complex conditions with multiple treatment options.

## Methods

This is a retrospective cohort study. All new patients referred from the skull base MDT and seen in the combined VS clinic from June 2013 to January 2019 inclusive were included. Coronavirus disease (COVID-19) affected the ability to run the clinic in its original format between 2019 and 2022 and therefore this time period was not included.

Patients that are suitable for both stereotactic radiosurgery (SRS)/radiotherapy or microsurgery are referred to the combined VS clinic via the skull base MDT and have a one-stop balanced consultation with a skull base surgeon, a skull base oncologist, and a skull base specialist nurse in the same sitting. Some of the patients with growing VS were offered WWR as one of the possible options because the growth was not beyond that which would be deemed acceptable over a 12-month period. The implications of tumor growth were considered in the clinical context, including future suitability for SRS with further growth, risk to facial function from subsequent delayed treatment, trigeminal neuralgia or risk of neuropathy following further growth and delayed treatment, hearing preservation, risk of mass effect on surrounding structures and patient choice after appropriate counseling. The main criteria for deciding if the patient was appropriate for referral to the combined VS clinic was suitability for either treatment option (SRS/Surgery) in patients who had a growing VS or new diagnosis at a size where further growth would cross a risk boundary for subsequent treatment. The final decision on whether the patient was appropriate for the combined clinic had to be triggered by consensus referral from the MDT meeting.

Electronic patient records, the picture archive and communication system and the national VS registry were accessed to collect data on patient demographics along with treatment decisions recommended by the MDT/made by patients over this time frame. Patients with recurrent or previously treated VS were excluded from the analysis.

Descriptive statistics and frequency analysis were carried out for the full cohort. Data from soon after the clinic was established in 2013–2014 were compared with later data from 2015 to 2019 to ascertain whether as the clinic became more established decision-making changed.

Ethics approval was obtained from the Health Research Authority through the University of Manchester (REC reference:20/NW/0015). In line with recommendations from the consensus meeting on systems for reporting results in acoustic neuroma,^17^ tumor size was measured as maximal length along the IAM (Internal Acoustic Meatus) for intracanalicular tumors and the maximal diameter of the cerebellopontine angle component for extra canalicular tumors, respectively.

## Results

Three hundred and fifty-four patients were included in the analysis. 91.3% of patients were referred to the combined clinic due to a growing VS; 8.7% due to the size of the VS at presentation. A total of 12% of VS were Koos grade I at presentation, 76% Koos grade II, 11% Koos grade III, and 1% Koos grade IV.

After face-to-face review in the interdisciplinary clinic, 309 patients (87%) were considered to be suitable for SRS or microsurgery. A total of 134 (37.9%) patients opted for SRS, 116 (32.8%) for conservative management, 94 (26.6%) for surgery, 4 (1.1%) for fractionated radiotherapy, 2 (0.6%) were discharged, and 3 (0.8%) patients asked for more time to make a decision at the time of the study. 95% of all cases referred to the joint VS clinic over this time period presented with tumors less than 25 mm in cerebellopontine angle diameter. This meant that other factors rather than purely size were taken into account when recommending treatment options to patients, since all patients were technically suitable for SRS or microsurgery.

**Figure 1.**
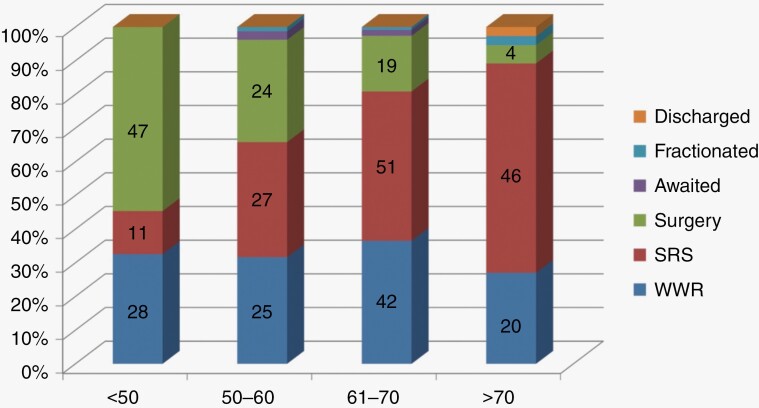
Treatment decisions stratified by age.

**Figure 2.**
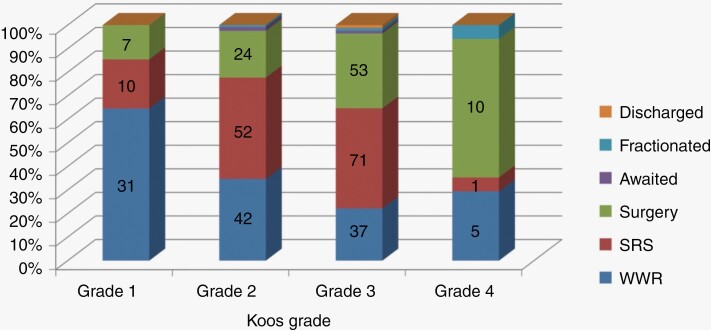
Treatment decisions stratified by Koos grade.

Figure 1 represents treatment decisions stratified by age. Fairly consistent numbers of patients of all ages opted for a WWR approach; with relatively high numbers of patients opting for surgical intervention as opposed to SRS. In those over the age of 70 only small number of patients elected for surgery with 62% of patients choosing to have SRS. The sex of the patient did not appear to play a significant role in decision making with 31% of women and 26% of men for all tumor sizes and patients ages electing to undergo surgery. Figure 2 shows treatment decisions based on Koos grade.

In contrast to the 2013–2014 data where no ASA (American Society of Anesthesiologists) 3 patients were offered surgery or stereotactic radiosurgery (SRS), the 2015–2019 data revealed that 10 out of 35 ASA 3 patients were offered SRS and 4 patients were offered surgery. Only 2 patients were discharged after review in clinic as they were deemed too unwell for monitoring or intervention (1 patient with rapidly progressive dementia and 1 patient was ASA 4).

**Figure 3.**
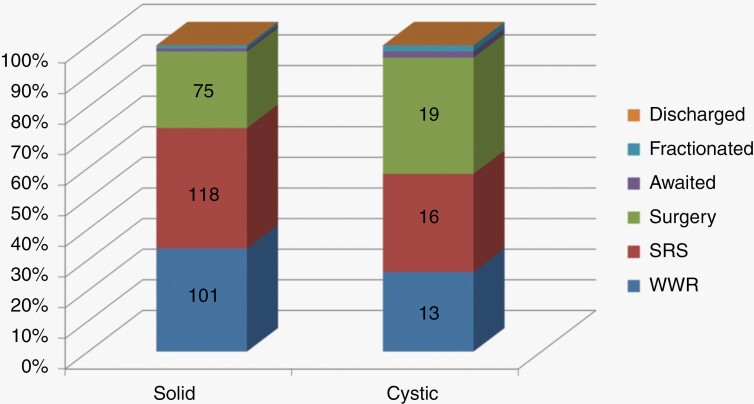
Treatment decisions solid vs. cystic tumor.

Of those with cystic tumors, 38% of patients underwent surgery compared to 25% of those with solid tumors. Conversely 39% of those with solid tumors underwent SRS versus 32% of those with cystic tumors (Figure 3). Cystic tumors were found to have a mean growth rate of 3.77 ± 3.10 mm/year (mean ± SD) vs. 2.91 ± 2.51 mm/year in solid tumors.

**Figure 4.**
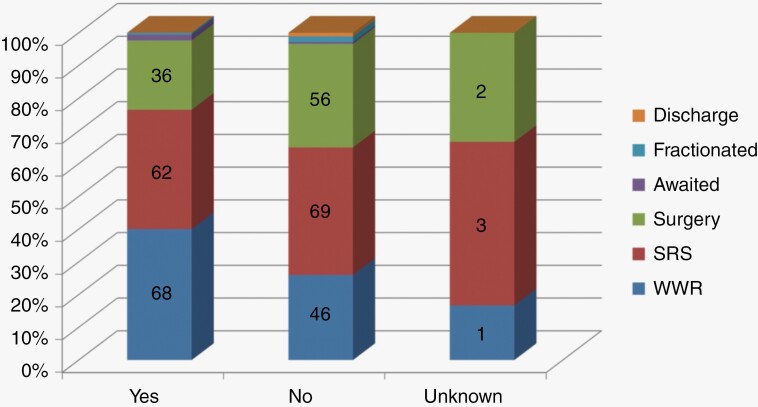
Treatment decisions stratified by useful hearing.

In those with useful hearing (Gardner-Robertson grade I and II) only 21% underwent surgical intervention with 44% of those without serviceable hearing (Gardner-Robertson grade III, IV, and V) electing to undergo surgery (Figure 4). Six patients (1.7%) had unknown information about their hearing status before being reviewed in clinic.

In this cohort, intracanalicular tumors grew on average at a rate of 2.09 ± 1.66 mm/year and extracanalicular tumors grew at an average rate of 3.18 ± 2.74 mm/year.

**Figure 5.**
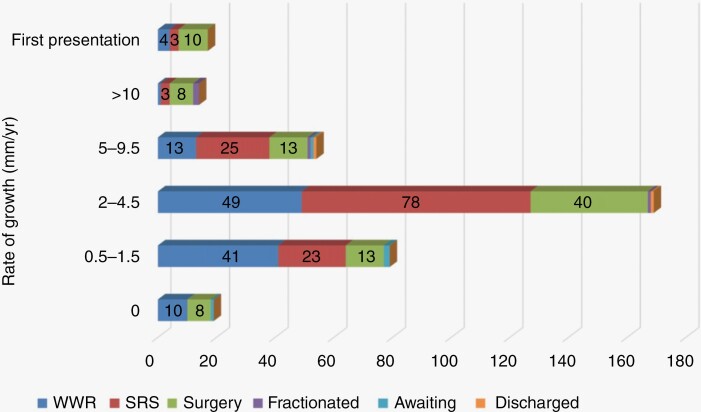
Treatment decisions stratified by rate of growth.

**Figure 6.**
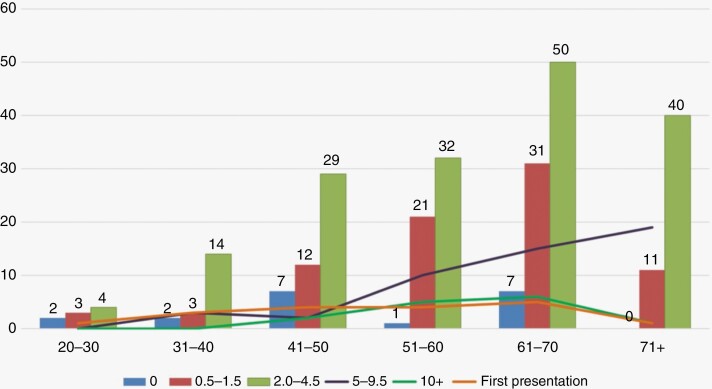
Rate of growth (mm/year) stratified by age.

A total of 65% of patients with a growing intracanalicular tumor were treated conservatively with a watch, wait and rescan approach. Of those with tumors growing at 1.5 mm per year or less only 21% underwent surgical treatment compared with 27.4% of those growing at 2 mm/year or greater (Figure 5). In our series, the fastest rates of growth were in those over the age of 71 years with relatively slow growth in those under 30 years of age (Figure 6). The under 30 group did, however, have very small patient numbers and therefore the generalizability in this age group should be interpreted with caution. Only 5% of patients reviewed in combined clinic had a VS that was not growing. The reasons for referral in this group are size approaching a risk boundary, trigeminal neuralgia, and patient request.

## Discussion

A variety of specialties have reported that multidisciplinary clinics where a group of healthcare providers with expertise in different areas of care manage complex medical conditions improve outcomes and are cost-effective.^18–20^ Sadiq et al.^21^ describe their “one stop shop” facial nerve clinic and found that combining appointments with multiple specialists was more convenient and reduced costs and wasted time for patients. Mclaughlin et al.^22^ further elaborated on the benefits of such a team approach not only to the patients in terms of patient satisfaction, improved efficiency, and delays in treatment while waiting for other speciality appointments but also to other healthcare providers (hospital trusts within our national health service) in terms of fewer canceled appointments and better interdisciplinary communication and learning. This approach is invaluable in patients with skull base pathology who otherwise may need to attend several appointments with ENT, audiology, neurosurgery, and neuro-oncology if all treatment possibilities were to be fully explored. It has been previously described in the literature how about a fifth of patients deciding how to manage their VS experience a significant degree of decisional conflict.^23^ Graham et al. described how SDM significantly reduced the uncertainty.^23^ Similarly, Diaz et al. describe how there is direct relationship between the quality of information received during the decision-making process and the level of anxiety of patients with high-grade gliomas.^24^

Healthcare professionals tend to agree that SDM is important for patients, but the practical applications of SDM are more challenging and modalities vary across the board.^25^ We described the model of our combined skull base and neuro-oncology clinic for VS featuring access to neurosurgery, ENT, and neuro-oncologists in a single appointment along with skull base SN and audiology. At the skull base MDT, patients with VS that are only suitable for one treatment are referred to the specific clinic, whilst patients amenable to more than one treatment option are referred to the combined VS clinic. A unique feature of this clinic is the semi-adversarial interdisciplinary counseling process with a surgeon presenting the advantages and disadvantages of surgery, an oncologist for radiosurgery or radiotherapy and a specialist nurse advocating for the patient and helping them to ask relevant questions and gain the relevant information to come to a shared decision with the team. Specialist nurses have a fundamental role as patient advocates and improve patients’ experience and overall care.^26^ We support this view and in our experience having a skull base SN present during the consultation helps patients in making a balanced decision and keeps them at the center of the decision-making process. In our experience, decision-making in this population of patients is more complex than expected and when presented with all treatment options patients do not necessarily choose the least invasive option as a treatment.

Since the combined VS clinic has been established relatively large numbers of patients have benefitted from its presence. Over time increasing numbers of medically more challenging patients have been referred for SRS or for surgical treatment as experience both surgically and anesthetically has developed in our high-volume center. In our cohort, roughly one-third of patients fall into each of the treatment strategies with slightly smaller numbers of patients undergoing surgery than WWR and SRS (26.6% vs. 32.8% and 37.9%, respectively). These figures are vastly different from those 25 years ago when almost all VSs were treated surgically. On a national level in 2011 the British skull base society described a national move towards watch, wait and rescan (on average 69% of patients) and a decrease in those treated surgically (on average 19%)^27^ with progressively increasing numbers of patients choosing to undergo SRS. This trend is likely to have continued to increase over time as availability of SRS has increased, as in our center, from 2011 onwards.

Leu et al.^28^ describe how healthcare staff satisfaction also increased with training and implementation of SDM. They suggest using decision grids as tools in the SDM process, a method also supported by Elwyn et al.^29^ We intend to develop option grids for decision-making for VS in our combined VS clinic as we believe they would increase patient understanding and guide healthcare staff.

## Conclusion

The combined surgery/oncology/SN clinic streamlines the patient experience for those with a VS suitable for either microsurgical or SRS/radiotherapy treatment. Decision-making in this population of patients is more complex than expected and when presented with all treatment options patients do not necessarily choose the least invasive option as a treatment. Some patients also decide to continue watching a growing tumor based on the risk discussed in clinic, the subsequent treatment implications, and their current holistic life situation. The unique feature of our clinic is the interdisciplinary counseling process with a surgeon presenting the case for surgery, an oncologist for radiosurgery or radiotherapy, and a specialist nurse advocating for the patient. Treatment options are likely to become more rather than less complex in future years making combined clinics more valuable than ever in SDM.
