# Corrigendum to: Surgery versus radiosurgery for vestibular schwannoma: Shared decision making in a multidisciplinary clinic

**DOI:** 10.1093/noajnl/vdae011

**Published:** 2024-02-03

This is a corrigendum to: Francesca Colombo, Helen Maye, Scott Rutherford, Andrew King, Charlotte Hammerbeck-Ward, Gillian A Whitfield, Catherine McBain, Rovel Colaco, Helen Entwistle, Andrea Wadeson, Simon Lloyd, Simon Freeman, Omar N Pathmanaban, Surgery versus radiosurgery for vestibular schwannoma: Shared decision making in a multidisciplinary clinic, Neuro-Oncology Advances, Volume 5, Issue 1, January-December 2023, vdad089, https://doi.org/10.1093/noajnl/vdad089

The data has been corrected/clarified as follows for the corresponding figures listed (corrections in italics):

**Table:** 

	Number				Total
All patients	*353*				*353*
	**Age**				
**Treatment decision (Fig. 1)**	**<50**	**50-60**	**61-70**	**>70**	
Surgery	47	24	19	4	94
SRS	11	27	*50*	46	*134*
WWR	28	25	42	20	115
Others*	0	3	3	4	10
**Solid Vs cystic tumor (Fig.3)**	**Surgery**	**SRS**	**WWR**	Others*	
Solid	75	118	101	8	302
Cystic	19	16	13	2	50
Unknown (no scan available)	0	0	1	0	1
**Useful hearing (Fig. 4)**	**Surgery**	**SRS**	**WWR**	Others*	
Yes	36	62	68	5	171
No	56	69	46	5	176
Unknown	2	3	1	0	6
**Rate of growth (mm/year) (Fig. 5)**	**Surgery**	**SRS**	**WWR**	Others*	
First presentation	*11*	3	4	0	18
0	8	*2*	10	1	21
0.5-1.5	13	*24*	41	1	79
2-4.5	40	78	*48*	2	168
5-9.5	14	*24*	*11*	4	53
>10	8	3	1	2	14
Total	94	134	115	10	353
**Koos grade** ^§^	**Surgery**	**SRS**	**WWR**	Others*	
Koos I	5	8	30	0	43
Koos II	66	118	76	7	267
Koos III	19	8	9	3	39
Koos IV	4	0	0	0	4
Total	94	134	115	10	353

*Others include: four patients for fractionated radiotherapy; two were discharged; three patients asked for more time to decide at the time of the study. One patient initially opted for conservative management but then had a duplicate attendance in clinic and proceeded to radiosurgery at their local center.

^§^ *Figure 2 should state Jackler Stage rather than Koos. Jackler 1 (1-10mm); 2 (11-20 mm); 3 (21-30 mm); 4 (31-40 mm). No giant VS in our series.*

In paragraph three of Results, “Fairly consistent numbers of patients of all ages opted for a WWR approach; with relatively high numbers of patients opting for surgical intervention as opposed to SRS” can be clarified as “Fairly consistent numbers of patients of all ages opted for a WWR approach; with relatively high numbers of patients under 50 years opting for surgical intervention as opposed to SRS.”

The revisions made do not change the outcomes or messages of the paper, nor the conclusions stated therein.

